# Rapid responses of human pluripotent stem cells to cyclic mechanical strains applied to integrin by acoustic tweezing cytometry

**DOI:** 10.1038/s41598-023-45397-5

**Published:** 2023-10-21

**Authors:** Zhaoyi Xu, Shiying Liu, Xufeng Xue, Weiping Li, Jianping Fu, Cheri X. Deng

**Affiliations:** 1 Department of Mechanical Engineering, University of Michigan, Ann Arbor, MI 48109 USA; 2 Department of Biomedical Engineering, University of Michigan, Ann Arbor, MI 48109 USA; 3 Department of Cell and Developmental Biology, University of Michigan Medical School, Ann Arbor, MI 48109 USA

**Keywords:** Biophysics, Stem cells, Engineering

## Abstract

Acoustic tweezing cytometry (ATC) is an ultrasound-based biophysical technique that has shown the capability to promote differentiation of human pluripotent stem cells (hPSCs). This study systematically examined how hPSCs respond to cyclic mechanical strains applied by ATC via displacement of integrin-bound microbubbles (averaged diameter of 4.3 µm) using ultrasound pulses (acoustic pressure 0.034 MPa, center frequency 1.24 MHz and pulse repetition frequency 1 Hz). Our data show downregulation of pluripotency marker Octamer-binding transcription factor 4 (OCT4) by at least 10% and increased nuclear localization of Yes-associated protein (YAP) by almost 100% in hPSCs immediately after ATC application for as short as 1 min and 5 min respectively. Analysis of the movements of integrin-anchored microbubbles under ATC stimulations reveals different stages of viscoelastic characteristic behavior and increasing deformation of the integrin-cytoskeleton (CSK) linkage. The peak displacement of integrin-bound microbubbles increased from 1.45 ± 0.16 to 4.74 ± 0.67 μm as the duty cycle of ultrasound pulses increased from 5% to 50% or the duration of each ultrasound pulse increased from 0.05 to 0.5 s. Real-time tracking of integrin-bound microbubbles during ATC application detects high correlation of microbubble displacements with OCT4 downregulation in hPSCs. Together, our data showing fast downregulation of OCT4 in hPSCs in respond to ATC stimulations highlight the unique mechanosensitivity of hPSCs to integrin-targeted cyclic force/strain dependent on the pulse duration or duty cycle of ultrasound pulses, providing insights into the mechanism of ATC-induced accelerated differentiation of hPSCs.

## Introduction

Human pluripotent stem cells (hPSCs), including both human embryonic stem cells (hESCs) and induced pluripotent stem cells (hiPSCs), have innate properties of unlimited self-renewal and pluripotency to differentiate into any type of specialized cells within adult body^1–3^. Thus, hPSCs are promising cell source for in vitro modeling of early development and pathological processes/diseases, as well as for cell-based therapeutics for a myriad of diseases. In conventional hPSC cultures, soluble factors (e.g., morphogens, growth factors and signaling pathway inhibitors) are supplemented in culture medium for directed differentiation of hPSCs into desired lineages^4–7^. However, current hPSC differentiation protocols are still suboptimal with poorly defined culture conditions, low differentiation purity and yield, as well as prolonged culture processes^8,9^. For example, using recently reported motor neuron (MN) differentiation protocols, oligodendrocyte transcription factor 2 (OLIG2) + MN progenitor cells and homeobox 9 (HB9) + MNs only appear in a hPSC culture on day 12 (purity < 80%) and day 21 (purity < 60%), respectively^7,10–13^. Thus, new technologies are needed to improve hPSC differentiation efficiency and speed towards functionally specialized cell types in order to fully realize the full potential and promise of hPSCs for disease modeling, cell-based therapies, and regenerative medicine.

On the other hand, insoluble “solid-state” signals of the cell microenvironment, such as extracellular matrix (ECM) rigidity and externally applied mechanical forces, have been recognized to play important roles in embryonic development, organogenesis^14–16^, and hPSC fate decision^17–20^. Despite increasing recognition regarding the importance of mechanical cues on hPSCs, comprehensive understanding of how these signals in the microenvironment of hPSCs regulate their fate decisions remains elusive. There are also increasing interests in developing novel biophysical strategies to leverage the intrinsic mechanosensitivity of hPSCs to improve their differentiation towards clinically relevant cell lineages^16,21^.

Lipid-encapsulated gaseous microbubbles are image-enhancing contrast agents used in clinical diagnostic ultrasound imaging^22^. The large mismatch of acoustic impedance between liquid and gas results in efficient interaction of ultrasound with microbubbles, producing robust scattering of the incident ultrasound field^23^. Ultrasound interaction with microbubbles leads to pronounced volume pulsation or cavitation of microbubbles^24^. In addition, microbubbles experience a directional force known as the acoustic radiation force due to momentum transfer from the incident ultrasound field^25^. Acoustic tweezing cytometry (ATC) leverages the acoustic radiation force of an ultrasound field acting on molecule-attached microbubbles to achieve molecularly targeted forces applied to cells. Specifically, in ATC, microbubbles are functionalized with desired ligands, e.g., Arginine-Glycine-Aspartic (RGD), on the microbubble shell to permit physical attachment of bubbles to the cell surface receptors integrin. Application of ultrasound pulses displaces integrin-bound microbubbles without bubble detachment by the acoustic radiation force, thereby effectively exerting controlled mechanical strains to the integrin-cytoskeleton (CSK) linkage of the cells^25,26^. In our previous studies, we show that ATC applications produced robust mechanoresponses in a number of cell types^25–29^, such as increased CSK contractility^26,28^ and enhanced osteogenic differentiation of mesenchymal stem cells^29^, by cyclically displacing microbubbles anchored to integrins on cell surface.

In particular, we have demonstrated that applications of ATC-mediated mechanical forces targeted to integrins of hPSCs for 30 min reduced expression of pluripotency-related transcription factors Octamer-binding transcription factor 4 (OCT4) and Nanog homeobox (NANOG) and induced epithelial-mesenchymal transition (EMT) immediately after ATC application^28^. Treatment with neural induction medium (NIM) following 30 min ATC application further resulted in increased expression of Paired Box 6 (PAX6), an early neuroectodermal (NE) differentiation marker, as early as 6 h, and neural rosette-like structure formation as early as 48 h^30^. These results show that ATC-facilitated NE differentiation of hPSCs occurred significantly faster than conventional NE differentiation protocols that rely on soluble factors only, which normally takes about at least 7 days to obtain PAX6 + neural rosette structures^31,32^.

Although we have previously shown that ATC-induced mechanoresponses in hPSCs depended on focal adhesion kinase (FAK) and CSK tension previously^28,30^, how integrin-targeted cyclic force/strain in ATC is transduced to elicit hPSC responses remains incompletely understood. Robust responses of hPSCs to ATC-mediated cyclic forces to integrins for only 30 min is intriguing and suggest exciting potentials of ATC to promote hPSC differentiation. The goal of this study is to determine the minimum application duration of ATC for eliciting responses in hPSCs. By assessing the changes in OCT4 in hPSCs immediately and within 30 min after ATC treatment, we aim to identify the early mechanotransductive events that proceed the signaling cascades regulating gene expression and fate changes in hPSCs. As cellular responses to mechanical signals are time-sensitive processes that can change overtime, determination of immediate responses of hPSCs to ATC could help isolate initial reactions from later ones, providing insights to the sequence of events and mechanisms in hPSC mechanostransduction.

## Materials and methods

### hPSCs culture

H9 human embryonic stem cells (WA09, WiCell; NIH registration number: 0062) were maintained in a standard feeder-free culture system using mTeSR1 medium (STEMCELL Technologies) and lactate dehydrogenase-elevating virus (LDEV)-free hESC-qualified reduced growth factor basement membrane matrix Geltrex (Thermo Fisher Scientific; derived from Engelbreth-Holm–Swarm tumours similarly as Matrigel) in accordance with the manufacturer's guidelines. Each passage involved a visual inspection to confirm the absence of spontaneously differentiated, mesenchymal-like cells in the culture. Only hPSCs before P70 were used in this work. For ATC experiments, cells are seeded at a density of 15,000 cells cm^-2^ in mTeSR in a glass bottom dish (MatTek Corporation) coated with 1% Geltrex on day 0. ROCK inhibitor Y-27632 (10 µM; Tocris) is supplemented in culture medium to prevent dissociation-induced apoptosis. On day 1, mTeSR1 is replenished without Y-27362. SB-431542 (10 µM, Stem cell Technologies) is added to mTeSR1 medium to inhibit TGF-β signaling and disrupt the pluripotency-maintaining gene circuit in hPSCs^33^. ATC experiments are performed on day 2.

### Conjugation of microbubbles with hPSCs

Biotinylated microbubbles with a diameter of 4.5 µm (Advanced Microbubbles Laboratories LLC) are mixed with streptavidin (Thermo Fisher, 10 mg/mL) at a volume ratio of 20:1 for 1 h at room temperature then washed away with PBS (phosphate buffered saline; Thermo Fisher Scientific, pH 7.4, sterile-filtered) to remove excess streptavidin. The streptavidin coated microbubbles are then conjugated with RGD-biotin (cyclo [Arg-Gly-Asp-D-Phe-Lys (Biotin-PEG-PEG)], Peptides International, 2 mg/mL) at a volume ratio of 10:1 for 1 h at room temperature, followed by washing with PBS to remove excess biotin-RGD. RGD-microbubbles are diluted 10 times in mTESR medium, and 50 µL is added to the culture dish seeded with hPSCs. The small volume of mTESR medium containing RGD- microbubbles is used to cover the monolayer of hPSCs and remain in place by capillary force when the culture dish is flipped upside down to allow microbubbles floating upwards to bind integrins on cell surfaces during 10 min incubation at 37 °C. Unbounded microbubbles are then washed away using PBS, and hPSCs with attached microbubbles are ready for ATC experiments (Fig. S1).

### ATC application

As described previously^28^, our ATC system uses a single element planar ultrasound transducer (Advanced Devices, Inc.) with an active radius of 0.635 mm. Two waveform generators (33220A; Agilent, Santa Clara, CA) and a 75 W power amplifier (75A250; Amplifier Research, Souderton, PA) are utilized to drive the transducer to produce ultrasound pulses for ATC applications. Before experiments, the ultrasound transducer is calibrated in a free field using a hydrophone (HNR-0500; Onda, Sunnyvale, CA). Hydrophone measurements confirmed that the transducer had a center frequency of 1.25 MHz, a Rayleigh distance or natural focal depth of 9.0 mm, and a − 6 dB beam width of 3.54 mm at the focal plane. The ultrasound transducer was positioned at 45° relative to a horizontally placed glass-bottom dish, to minimize the artifact of standing wave formation within the dish while permitting unobstructed microscopic imaging. The active surface of the transducer was submerged in the medium and 9.0 mm away from cells, at the Rayleigh distance. The acoustic pressure of the pulses was kept at 0.034 MPa, and the pulse repetition frequency (PRF) was kept at 1 Hz, while the duty cycle was selected to be either 5%, 25%, or 50%, as described in various experiments to actuate integrin-bound microbubbles.

### Monitoring and characterization of ultrasound-driven microbubbles

Movements of microbubbles during ATC applications are recorded using an inverted microscope (Nikon Eclipse Ti-U) and a high-speed camera (Photron FASTCAM SA1) operated at 1000 frames per second and a subpixel resolution of 330 nm. Tracking of individual microbubbles was performed using an algorithm previously developed^25^. To track displacements of multiple microbubbles simultaneously during ATC application, a new Python-based algorithm is developed to identify and determine dynamic microbubble movements from recorded videos. Python package ‘Trackpy’ (v0.5.0), based on the widely used Crocker-Grier algorithm for tracking blob-like objects^34^, is employed to quantify bubble size and displacement as a function of time. The cumulative displacement of each bubble is determined as the total movement of the bubble during the observation time period. The area under the curve (AUC) for bubble movement is calculated by integrating the bubble displacement curve (y-axis values) across the observation time window (x-axis values), consistent with our prior research^28^.

### Immunocytochemistry analysis to detect changes due to ATC treatment

After ATC experiments, cells are fixed with 4% paraformaldehyde (PFA; buffered in PBS, Electron Microscopy Sciences) for 30 min and permeabilized with 0.1% sodium dodecyl sulfate (SDS; buffered in PBS, Sigma-Aldrich) for another 30 min. After incubation with 4% donkey serum (buffered in PBS, Sigma-Aldrich) for 1 h to block non-specific binding, cells are incubated with primary antibodies against YAP (Yes-associated protein) (YAP (D8H1X) XP Rabbit mAb #14074, Cell Signaling Technology) or OCT4 (Oct3/4 Antibody (C-10): sc-5279, Santa Cruz Biotechnology) for 1 h, and then for another 1 h with secondary antibodies. Cell nuclei are counterstained with 4,6-diamino-2-phenylindole (Invitrogen™ DAPI (4',6-Diamidino-2-Phenylindole, Dihydrochloride) GSA_VA, Thermo Fisher Scientific) for 1 h together with secondary antibodies. Staining for NANOG are performed with similar approaches (Nanog (D73G4) XP® Rabbit mAb #4903, Cell Signalling Technology). After staining, fluorescence images are taken using an Olympus DSUIX81 spinning disc confocal microscope equipped with an EMCCD camera (iXon X3, Andor). Expression level of OCT4 in hPSCs is determined by quantifying immunofluorescent intensity of OCT4 in cell nuclei, a common practice in hPSC research^35,36^. Quantification of nuclear fluorescent intensity is performed using ImageJ (NIH). Specifically, nuclear OCT4 intensity is determined through the following process: Firstly, DAPI channel images are converted to 8-bit. A threshold is then automatically determined from average background signal to demarcate nucleus region and determine nuclear signal intensity relative to average background signal. After applying binary masking, OCT4 channel images are obtained, and nuclear OCT4 intensity is determined. To address variations in staining at different batches, OCT4 intensity values are normalized to average DAPI intensity for each image. YAP immunostaining images are also analyzed using ImageJ. Cells with 10% higher nuclear YAP fluorescence intensity than the cytoplasmic fluorescence intensity are identified as cells with positive nuclear YAP.

### Statistics

For each group/condition, experiments are repeated for at least three times, with each time using different batches of cells. For analyzing data with a normal distribution, two-sample *t*-test is performed. Nonparametric Mann–Whitney test is performed for data without a normal distribution. Differences are deemed statistically significant when *P* < 0.05.

## Results and discussions

### ATC applies mechanical strains to hPSCs by displacing integrin-bound microbubbles

**Figure 1:**
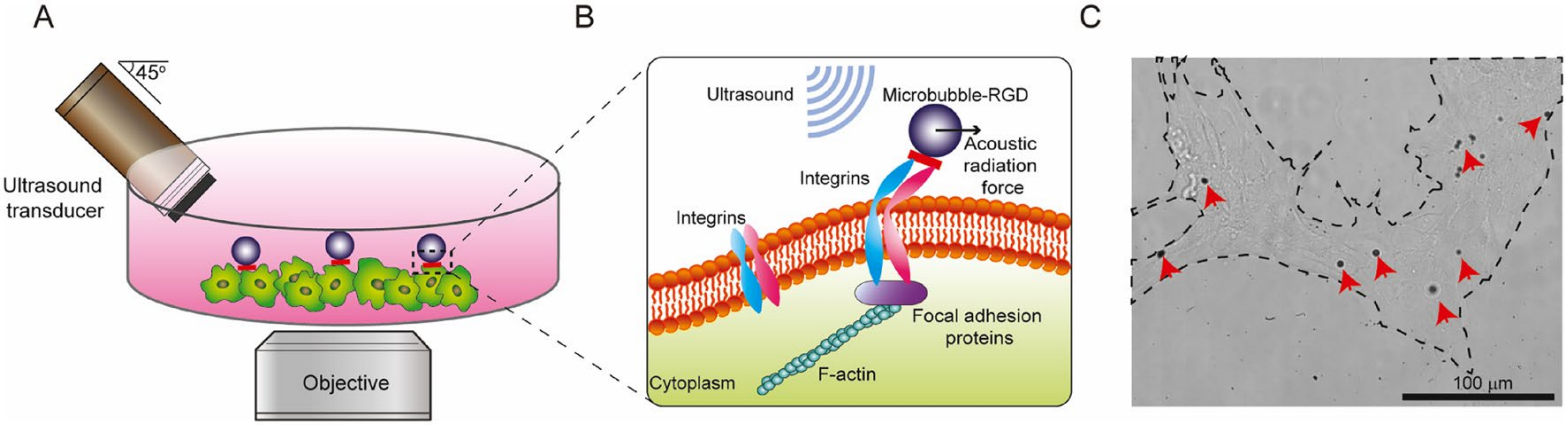
(**A**) Schematic for acoustic tweezing cytometry (ATC) integrated with microscopic imaging, where the transducer was positioned as a 45° angle to avoid standing wave formation. (**B**) Integrin-anchored microbubbles functionalized with Arg-Gly-Asp (RGD) were subjected to acoustic radiation force associated with an ultrasound field. (**C**) Representative bright field image of a human embryonic stem cell (hPSC) colony with attached microbubbles (red arrowheads).

As shown in Fig. 1, ATC uses an ultrasound transducer to generate ultrasound pulses to displace microbubbles that are attached to integrins on the membranes of hPSCs. For experiments in this study, the transducer was oriented at a 45° angle with respect to the vertical axis to avoid standing wave formation in the cell culture dish while permitting microscopic imaging of hPSCs and microbubble dynamics (Fig. 1A). Because of the acoustic impedance mismatch between fluid and gas, the acoustic radiation force generated by incident ultrasound field is significantly greater on microbubbles than on surrounding medium fluids or hPSCs. As a result, applied ultrasound pulses establish a net vector force acting on integrin-bound microbubbles to push them in the ultrasound field propagating direction, while having negligible effects on other components of the cells within the broad ultrasound field. The first-order estimate of the primary acoustic radiation force *F* on a microbubble of radius *R*_*0*_ by an ultrasound field with acoustic pressure *P*_*A*_ and angular frequency ${\omega }_{0}$ can be calculated from the following equation^25,26,37^,

$$F\cong \frac{2\pi {P}_{A}^{2}{R}_{0}}{{\delta }_{tot}{\rho }_{0}c{\omega }_{0}}$$

where the total damping constant is ${\delta }_{tot}\sim 0.16$, the medium density ${\rho }_{0}=1000$ kg m^−3^, and speed of sound $c=1500 m {s}^{-1}$. In our experiments with an ultrasound field at 1.25 MHz and 0.035 MPa, the acoustic radiation force exerting on microbubbles with a radius of 1.5–2.0 µm was calculated to be about 17.0–25.0 nN, as the result of the primary acoustic radiation force due to a plane ultrasound field at 1.25 MHz and acoustic pressure of 0.034 MPa^25,26,37^ Displacement of integrin-bound microbubbles under ATC applications stretches and deforms the bubble-integrin-CSK linkage, therefore generating mechanical strains in the molecular complex of the integrin-CSK linkage (Fig. 1B). Displacement of an integrin-bound microbubble depends on acoustic pressure, duration of ultrasound pulse (duration of force application), bubble size, and mechanical property (e.g. stiffness) of the bubble-integrin-CSK linkage.

Ultrasound waves have relatively long wavelengths compared to dimensions of cells. Thus, ultrasound waves are typically unable to apply targeted forces at molecular or cellular scales. For example, ultrasound wavelength is about 1.25 mm for ultrasound at a frequency of 1.0 MHz, which is several orders of magnitude larger than mammalian cells (< 50 µm). In contrast, the acoustic radiation force acting on microbubbles is significantly greater than the force on a single cell, owing to robust interaction of ultrasound with gaseous bubbles as the result of large acoustic impedance difference between gas and surrounding medium solution. By using functionalized micron-sized microbubbles bound to cell surface receptors such as integrins, ATC is able to focus force applications on bubbles, thereby generating mechanical strains to molecular targets on cell membranes by displacing integrin-anchored bubbles with a broadly applied ultrasound field, useful for treating a large number of cells with attached bubbles simultaneously (Fig. 1C).

As shown in Fig. 1C, in this work microbubbles were sparsely attached to top surfaces of hPSCs seeded on culture dishes. Bubble to cell ratio in ATC experiments can be readily controlled by adjusting microbubble concentrations in our experiments. While higher concentrations of bubbles might result in more efficient responses of hPSCs, we chose a concentration of bubbles that yielded a bubble:cell ratio of about 1:10 to minimize potential damages to hPSCs due to sonoporation^38^ or the impact of cavitation as shown in our previous studies^24,38,39^. Importantly, our previous studies show hPSCs in a colony could respond globally to ATC applications, exhibiting increased CSK contractility and downregulation of pluripotency markers across the entire colony, even when not all cells are coated with microbubbles^28,30^. The global response of hPSC colonies likely results from mechanical and chemical interactions between adjacent hPSCs in the same colony^39^.

### ATC treatment induced rapid changes in hPSCs

OCT4 is a transcription factor essential for pluripotency maintenance of hPSCs, and OCT4 expression decreases in hPSCs when the cells start to differentiate^40^. In our experiments, OCT4 expression is used as a faithful reporter of hPSC pluripotency. We have shown previously that application of ATC for 30 min to hPSCs elicited immediate loss of their pluripotency with significantly reduced OCT4 expressions^28^. In the current study, we conducted a series of experiments aiming to determine the minimum ATC duration capable of initiating changes in hPSCs and their early responses to ATC treatment.

#### Downregulation of OCT4

**Figure 2:**
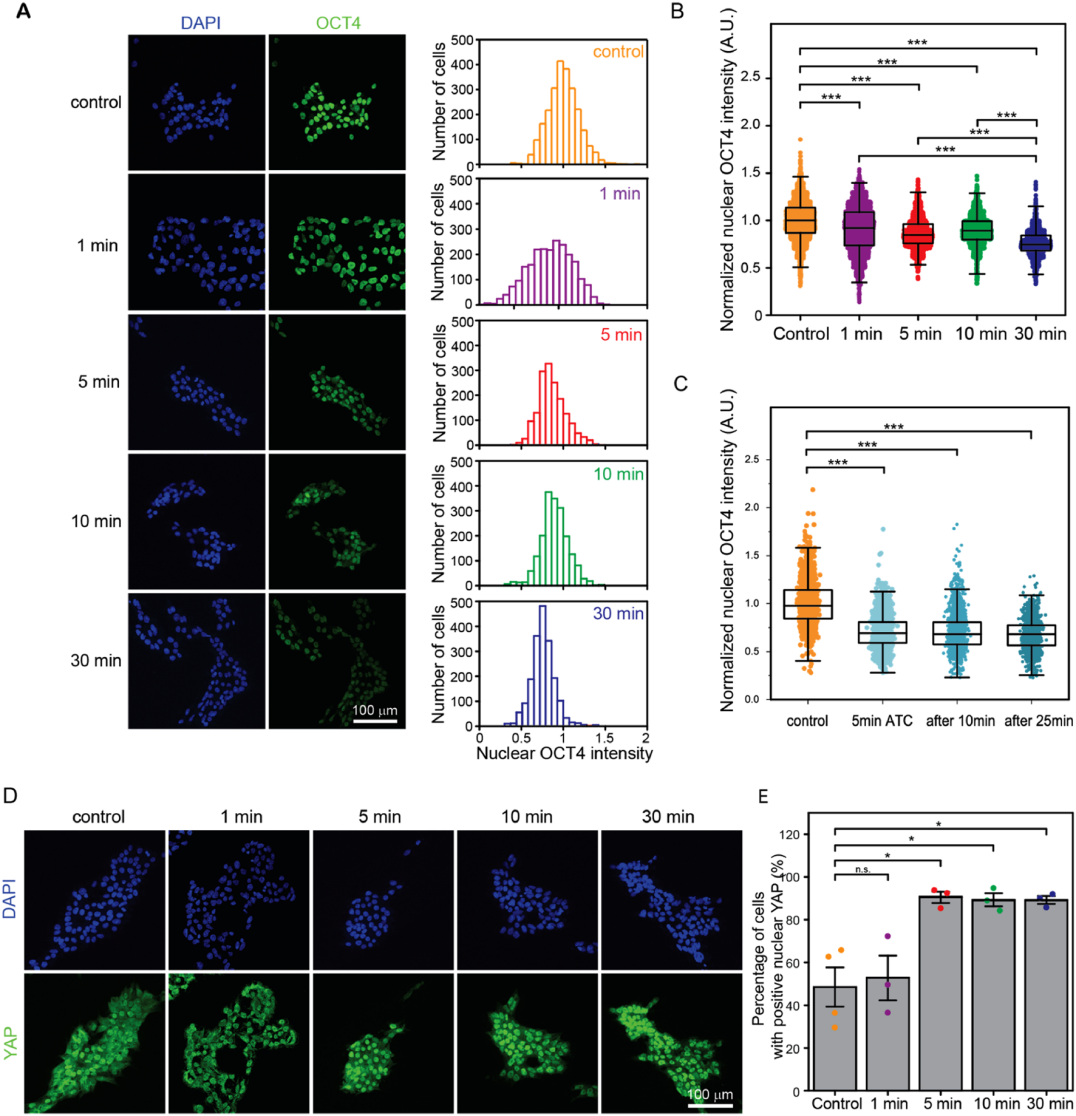
(**A**) Representative confocal microscopic images and histograms showing immunostaining of OCT4 in hPSCs with and without ATC treatment for different durations. hPSCs were stained at 30 min after the start of ATC application. (**B**) OCT4 fluorescence intensity in hPSCs with and without ATC treatment for different durations (box: 25–75%, bar-in-box: median, and whiskers: 1% and 99%). Cells were stained at 30 min after the start of ATC application. (**C**) OCT4 fluorescence intensity in hPSCs fixed and stained at different time points after the start of 5.0 min ATC treatment (box: 25–75%, bar-in-box: median, and whiskers: 1% and 99%). (**D**) Representative confocal microscope images showing immunostaining of YAP for untreated cells and treated hPSCs for different duration of ATC. (**E**) The percentage of cells with positive nuclear YAP. Data are expressed as mean ± s.e.m. *p < 0.05, **p < 0.01, ***p < 0.001.

In our experiments to determine responses of hPSCs to ATC stimulations, we used the same ATC parameters as in our previous studies, except that different ATC application duration was used in our current study^26–28,41^. Specifically, we applied ATC using ultrasound pulses at a 1.0 Hz pulse repetition frequency (PRF) and 50% duty cycle with an acoustic pressure of 0.035 MPa for different durations, i.e., 1 min, 5 min, 10 min and 30 min, respectively. hPSCs were fixed and stained for OCT4 30 min after the onset of ATC applications, regardless of ATC durations. Our data show that ATC application for as short as 1 min could result in notable downregulation of OCT4 in hPSCs, as shown by decreased mean values of OCT4 fluorescent intensity compared to those in control conditions (Fig. 2A,B). Interestingly, while the mean value of OCT4 fluorescent intensity did not decrease further in hPSCs treated with longer durations of ATC, the distribution range of OCT4 intensity in hPSCs shrank notably when ATC duration increased beyond 1 min (Fig. 2A,B).

To determine whether downregulation of OCT4 in hPSCs occurred immediately after ATC stimulations, we conducted another set of experiments in which ATC was applied for 5 min and hPSCs were fixed and stained at different time points after ATC treatments. Specifically, instead of fixing hPSCs 30 min after the onset of ATC treatments, hPSCs were fixed and stained 5 min (immediately after 5 min ATC treatments), 15 min, or 30 min after the onset of ATC applications, respectively. Our data show that downregulation OCT4 became evident immediately after ATC stimulations, and this OCT4 downregulation was sustained for at least another 25 min after ATC stimulations (Fig. 2C, Fig. S2).

Our data of rapid downregulation of OCT4 in hPSCs by ATC (as short as 1 min) show the early mechanoresponses of hPSCs, showing their unique mechanosensitivity to ATC stimulations and the likely role in the long-term effects on hPSCs after ATC treatments manifested as faster differentiation and neural rosette formation^28,30^.

#### Nuclear translocation of YAP

As shown in Fig. 2D,E, the percentage of hPSCs with positive nuclear YAP after 1 min ATC application (53% ± 11%, n = 3) show no significant change compared to control (50% ± 10%, n = 4). However, the percentage of hPSCs with positive nuclear YAP after ATC treatment for 5 min, 10 min, or 30 min increased significantly to 96% ± 4%, 95% ± 3%, and 95% ± 2%, n = 3 respectively. The decreased percentage range of positive nuclear YAP corresponds to our observation that OCT4 exhibited smaller ranges in cells subjected to ATC with duration longer than 5 min (Fig. 2C), indicating more uniform changes generated by longer ATC duration.

We further conducted experiments in which hPSCs were treated by ATC for 5 min but fixed at different times. Increased percentages of hPSCs with nuclear YAP were observed immediately after ATC treatment. Similar to downregulation of OCT4 by ATC stimulations, increase of percentages of hPSCs with nuclear YAP was sustained for at least of 25 min after 5 min ATC application (Fig. S3).

YAP activity in hPSCs has been suggested to be important for pluripotency maintenance^21^. YAP is also a known nuclear effector converting intracellular mechanotransducive signaling activities into gene regulation programs^42^. In a rigid environment, YAP signaling is activated in hPSCs, promoting YAP nuclear translocation to support pluripotency maintenance of hPSCs^43^. Modulation of YAP activity is necessary for hPSCs to exit pluripotency and begin differentiation into specific lineages^21,29,44,45^. Our observation of decreased OCT4 in hPSCs after ATC application is consistent with our previous results^28,30^, indicating that the hPSCs were undergoing differentiation due to ATC application. However, YAP nucleocytoplasmic translocation in hPSCs after 30 min ATC treatment was observed in our previous study^28^ rather than nuclear translocation observed in our current study. This discrepancy may be attributed to the differences in protocols. In our previous studies, ATC experiments were performed on hPSCs 1 day after cell seeding, whilst in our current study ATC treatments were applied to hPSCs on day 2 after cell seeding which enabled better cell attachment and viability. In addition, in our current protocol, on day 1, mTeSR1 was replenished without Y-27362 and SB-431542 (10 µM, Stem cell Technologies) was added to mTeSR1 medium to inhibit TGF-β signaling and disrupt the pluripotency-maintaining gene circuit in hPSCs^33^. ATC experiments were then performed on day 2. Thus the different observations indicate the sensitivity of YAP activity to culture protocols and stages of cells when the mechanical forces are applied. The roles of YAP/TAZ in stem cell fates have been shown to be context dependent^46^ and YAP could exhibit cell state-dependent nuclear re-entry to regulate lineage-specific genes after initial exit during commitment.

### Displacement of integrin-bound microbubbles affected by ultrasound pulse parameters

In ATC, an integrin-bound microbubble serves as the point of force application to a cell, providing mechanical signals to the cell through the integrin-CSK linkage. While application of ultrasound waves also generates effects such as microstreaming that may also impact cell behaviors, we have previously shown that ultrasound mediated displacement of integrin-anchored microbubbles in ATC is the main factor responsible for generating the observed changes in cells, compared to microstreaming or direct cell compression from ultrasound field^25^. To help gain better understanding of how ATC exerts its robust impact on hPSCs, we next conducted experiments to examine movement of integrin-bound microbubbles during ATC treatments.

Our previous studies have optimized acoustic pressure of ultrasound pulses for displacing integrin-bound microbubbles without negatively affecting cell viability by minimizing the effect of bubble cavitation that can cause sonoporation and/or mechanical lysis of cells^25^. However, how other ultrasound parameters affect movements of integrin-bound microbubbles has not been examined systematically. In this study, we focused on the effects of duty cycle of ultrasound pulses with a fixed PRF (1 Hz) and acoustic pressure (0.035 MPa). At PRF of 1 Hz, changing duty cycle from 5 to 25% and 50% increases the duration of each pulse from 0.05 to 0.25 s and 0.5 s, respectively, followed by an “off” period of 0.95–0.75 s and 0.5 s, respectively, before the next pulse. Thus, increasing duty cycle in ATC increases the duration of constant force application on integrin-microbubbles, which is followed by a shorter “off” period when the acoustic radiation force is turned off before the next pulse. Note even though the temporal average of the acoustic intensity was different for ultrasound pulses with different duty cycle, the bubble movements only depended on the acoustic pressure and the pulse duration.

#### Viscoelastic characteristics of ATC-induced displacement of integrin-bound microbubbles

**Figure 3:**
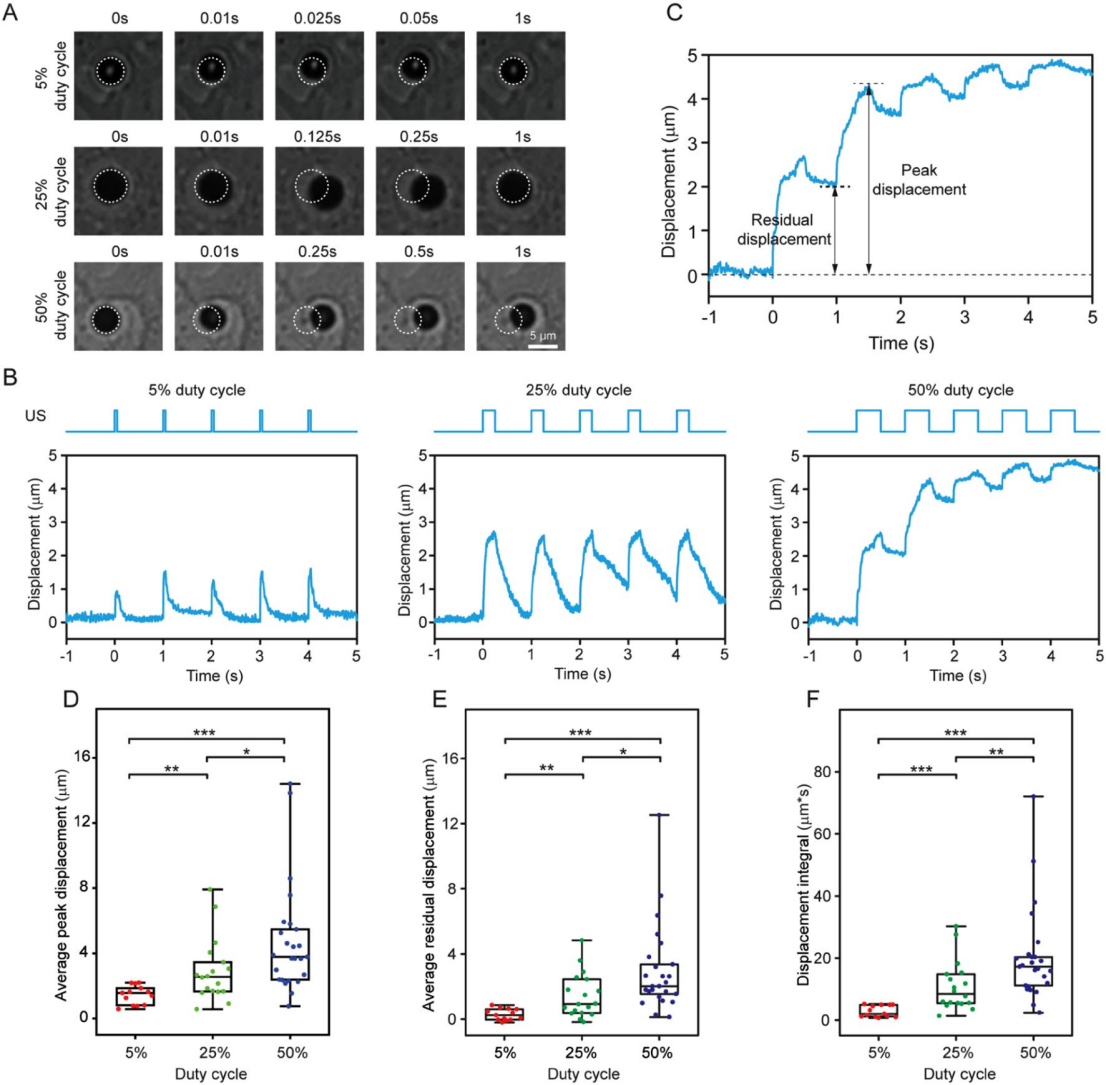
(**A**) Representative bright field images of microbubbles at specific time points actuated by ultrasound using different duty cycles (acoustic pressure 0.035 MPa). White circles represent the original positions of microbubbles before ultrasound pulse. (**B**) Microbubble displacement profiles recorded during the first five ultrasound pulses. (**C**) Schematics showing definition of peak and residual displacement respectively. (**D–F**) Average peak displacement (**D**), average residual displacement (**E**), and displacement integral (**F**). Box: 25–75%, bar-in-box: median, and whiskers: 1% and 99%. *p < 0.05, **p < 0.01, ***p < 0.001.

During an ultrasound pulse, integrin-bound microbubbles were subjected to a step force until the pulse was turned off, with the duration of force application determined by pulse duration. In response, integrin-bound microbubbles were pushed away from their original positions on cell membranes and retracted when the ultrasound pulse was turned off (see examples in Fig. 3A,B). Application of a series of ultrasound pulses (PRF 1 Hz) thus resulted in cyclic movements of integrin-bound microbubbles, with a repeated pattern of displacements and retractions matching the temporal sequence defined by the PRF and pulse duration/duty cycle of ultrasound pulses (Fig. 3B). Notably, dynamic movements of integrin-bound microbubbles exhibited the characteristics of a viscoelastic system that was influenced by duty cycle of ultrasound pulses (Fig. 3B). Even when the same acoustic pressure was used, ultrasound pulses with greater duty cycles generated greater bubble displacements that only recovered partially before the next ultrasound pulse (Fig. 3A,B). When subjected to ultrasound pulses at a duty cycle of 5%, integrin-bound microbubble movement exhibited a creep behavior before reaching a displacement distance of 1–2 µm during a period of 0.05 s. afterwards, microbubbles underwent a time-dependent recovery, moving towards their original positions (left panel, Fig. 3B). The dynamic movements of microbubbles, resembling the primary creep stage of a viscoelastic system and consistent with a linear viscoelastic *Kelvin-Voigt* model, indicate the viscoelastic nature of the bubble-integrin-CSK system. At duty cycle of 25%, microbubbles achieved greater displacements of 2–3 µm during the longer duration of ultrasound pulse application. Interestingly, a secondary creep stage appeared beyond the primary creep near the end of the longer ultrasound pulse at 0.25 s (middle panel, Fig. 3B). Recovery of microbubble displacement was partial, as microbubbles did not retract all the way back to their original positions after each ultrasound pulse, suggesting unrecovered deformation of the integrin-CSK linkage. When ultrasound duty cycle was increased to 50%, microbubbles achieved even greater displacements, to a level where a tertiary creep stage emerged by the end of the first ultrasound pulse at 0.5 s (right panel, Fig. 3B). This signifies microbubble displacement that remained unrecovered, suggesting changes generated in the integrin-CSK linkage with complex viscoelastic behaviors beyond the linear regime. Retraction of microbubbles, after a small instantaneous/elastic recovery, showed substantial unrecovered deformation (right panel, Fig. 3B), suggesting elevated plasticity and changes to the integrin-CSK linkage that continued to build up during subsequent ultrasound pulses.

#### Characterization of effects of duty cycle of ultrasound pulses on microbubble displacement

We defined and extracted several parameters, as illustrated in Fig. 3C, to characterize dynamic microbubble movements influenced by duty cycle of ultrasound pulses. The bubble peak displacement represents the maximum displacement a bubble achieves during each ultrasound pulse. The residual bubble displacement denotes unrecovered displacement of a bubble relative to its original position before the next ultrasound pulse, reflective of unrecovered deformation of the bubble-integrin-CSK linkage. The area under the curve (AUC) is determined by integrating bubble displacement over time, reflective of an “energy input” or “work done” to the system by ultrasound pulses applied during ATC.

As representative metrics, we calculated the average values of bubble peak and residual displacements measured during the first five consecutive ultrasound pulses. Our data show that the average bubble peak displacements were significantly different between microbubbles subjected to ATC with different duty cycles (Fig. 3D). For example, the average peak displacement was 1.45 ± 0.16 μm for 5% duty cycle, compared to 4.74 ± 0.67 μm for 50% duty cycle (Fig. 3D). The average residual displacements also increased, from 0.28 ± 0.10 μm at 5% duty cycle to 1.44 ± 0.32 μm and 2.97 ± 0.54 μm for 25% and 50% duty cycle respectively (Fig. 3E). The AUC increased from 2.81 ± 0.51 μm-s for 5% duty cycle, to 10.88 ± 1.88 μm-s and 20.28 ± 3.01 μm-s for 25% and 50% duty cycle respectively (Fig. 3F). Relatively large variations were seen in the results at greater duty cycles, likely due to non-linear irregularity associated with large deformation of the viscoelastic bubble-integrin-CSK linkage resulted from longer durations of ultrasound pulses.

### Simultaneous tracking of movements of multiple bubbles during ATC application

Our results reveal large variations in microbubble displacements during ATC treatment of hPSCs at a high duty cycle of 50% (Fig. 3). To gain a better understanding of such variations, we developed an algorithm to automatically track movements of multiple bubbles simultaneously to ascertain both the direction and magnitude of their movements during ATC.

**Figure 4:**
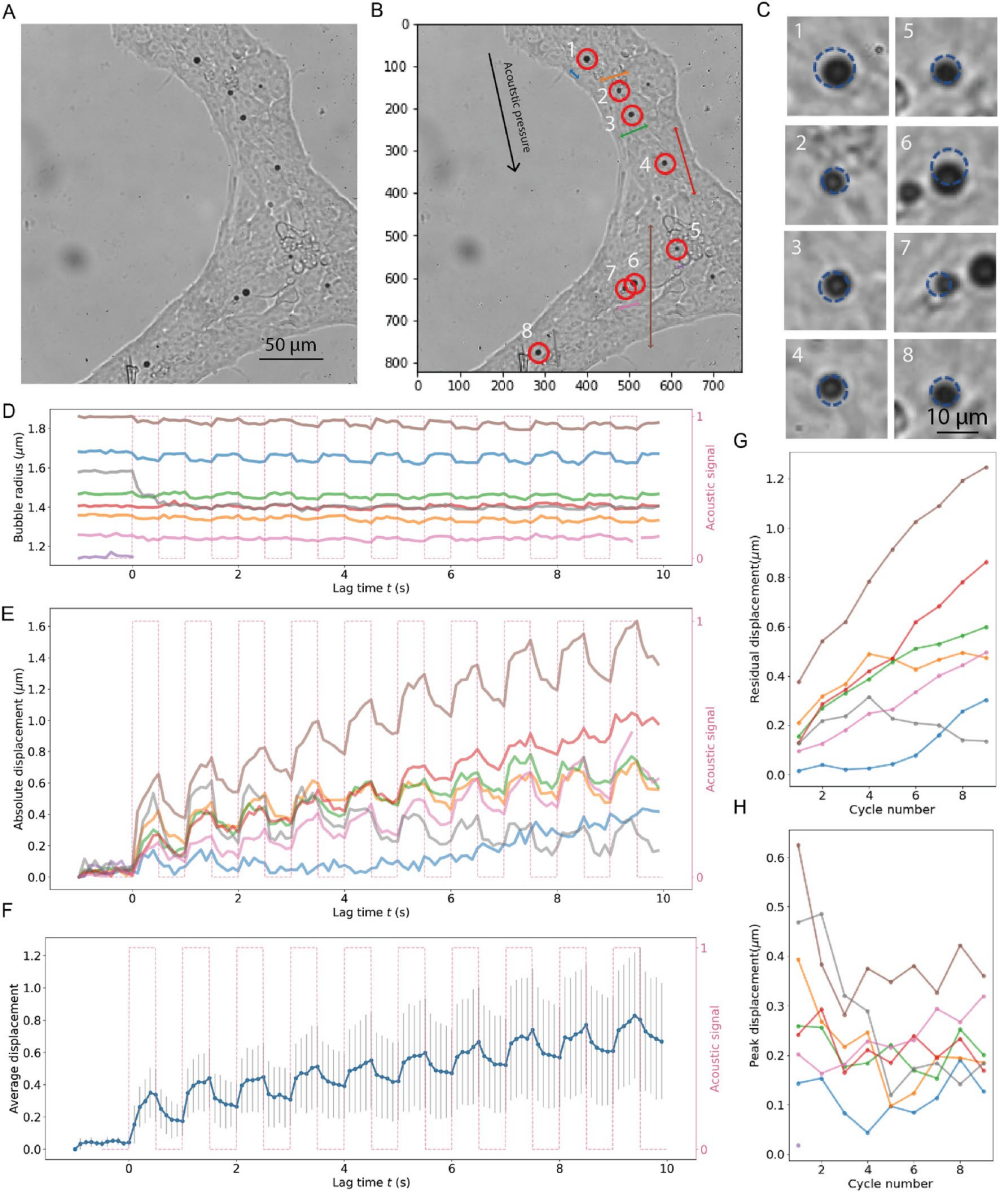
(**A**) An hPSC colony with sparsely attached microbubbles. (**B**) Custom bubble tracking algorithm detects movement of multiple microbubbles. The length and direction of the arrow represent the relative magnitude and direction of displacement vector for each bubble distinguished by different colors. (**C**) Images showing the displacement of bubbles during the first pulse. (**D**) The radius of the microbubbles in (**A**) as function of time. (**E**) The measured absolute displacement of the microbubbles in (**A**). (**F**) Average displacement of all microbubbles as a function of time. (**G**) The residual displacement of the microbubbles in (**A**) during the first 10 pulses for the bubbles in (**A**). (**H**) The difference of peak displacement and residual displacement during the first 10 pulses for the microbubbles in (**A**).

As shown in Fig. 4, integrin-bound microbubbles were sparsely attached to a hPSC colony during ATC experiments. Although hPSCs and microbubbles were situated under the same ultrasound field (Fig. 4A), movements of integrin-bound microbubbles were non-uniform and appeared to depend on their relative spatial locations with each other. Specifically, microbubble displacements not only differed in magnitude but also in direction (Fig. 4B,C), while the bubble size decreased only slightly (Fig. 4D) due to leaking of gas from the bubbles during cavitation. The phenomenon of bubble displacement in multiple directions strongly implies that, beyond the primary acoustic radiation force, which exerts a uniform force direction on all microbubbles in the direction of the incident ultrasound pulse, additional forces were present to influence microbubble movements. This is consistent with our previous results that show bubble–bubble interaction in the form of the secondary acoustic radiation force resulted from the scattering field of the incident ultrasound field by microbubbles nearby. As a result, a bubble experiences not only the primary acoustic radiation force generated by the incident ultrasound field but also the secondary acoustic radiation force, or secondary Bjerknes force. For a given incident ultrasound field (e.g. acoustic pressure and frequency) and the size of bubbles, the secondary Bjerknes force increases with the inverse of squared distance between two bubbles and is in the direction along the line connecting the two bubble centers^25^. Therefore the total acoustic radiation force acting on a bubble may not be in the same direction of the primary acoustic radiation force^25^, resulting in displacement of microbubbles in different directions even though they are subjected to the same primary ultrasound wave.

Besides the directionality of bubble displacements, the magnitude of displacements of integrin-bound microbubbles exhibited similar viscoelastic characteristics although with large variation (Fig. 4E,F), which could also be attributed in a large part to the impact of the secondary Bjerkes force in addition to the primary acoustic radiation force. In general, the residual deformation or unrecovered bubble displacement after each ultrasound pulse increased with additional pulses (Fig. 4G), suggesting cumulative strains and deformation in the integrin-CSK linkage within the cells induced by displacement of integrin-bound microbubbles. Interestingly, while the peak bubble displacement increased with subsequent ultrasound pulses, the relative displacement generated by each pulse, which is the difference between the peak displacement and the residual displacement for each pulse, stayed somewhat unchanged with much less variation for each individual bubble after the initial pulses (Fig. 4H). This observation suggests that ATC treatment may homogenize the integrin-CSK linkage within the cells so that the integrin-CSK linkage reached a stable state after the initial large deformation.

Taken together, the integrin-CSK linkage within hPSCs exhibited a viscoelastic characteristics with time-dependent strain response and recovery that depended on the duration of acoustic force application. ATC induced deformation to hPSCs via the integrin-CSK linkage that did not completely recovery at duty cycles of 25% and 50%, or pulse duration of 0.25 s and 0.5 s, respectively. Such large deformation might induce structural or conformation changes in the integrin-CSK linage, which might in turn expose protein binding / activity sites to trigger downstream mechanotransducive processes in hPSCs.

### Displacement of integrin-bound microbubbles correlated with changes in hPSCs

Displacement of integrin-bound microbubbles induced mechanical strains to hPSCs through the bubble-integrin-CSK linkage during ATC application. Here we quantified and correlated molecular changes in hPSCs with displacements of integrin-bound microbubbles in experiments using 30 min ATC application at different duty cycles (PRF 1 Hz). In these experiments, ultrasound pulses were applied with acoustic pressures of 0.035 MPa, 0.045 MPa, and 0.055 MPa for three consecutive 10 min for a total of 30 min ATC application. This is to compensate for the reduction in microbubble radius over time due to gas leakage during cavitation as described previously^28,30^. The compensation is needed because smaller microbubbles require higher acoustic pressure for displacements by acoustic radiation forces^47^.

**Figure 5:**
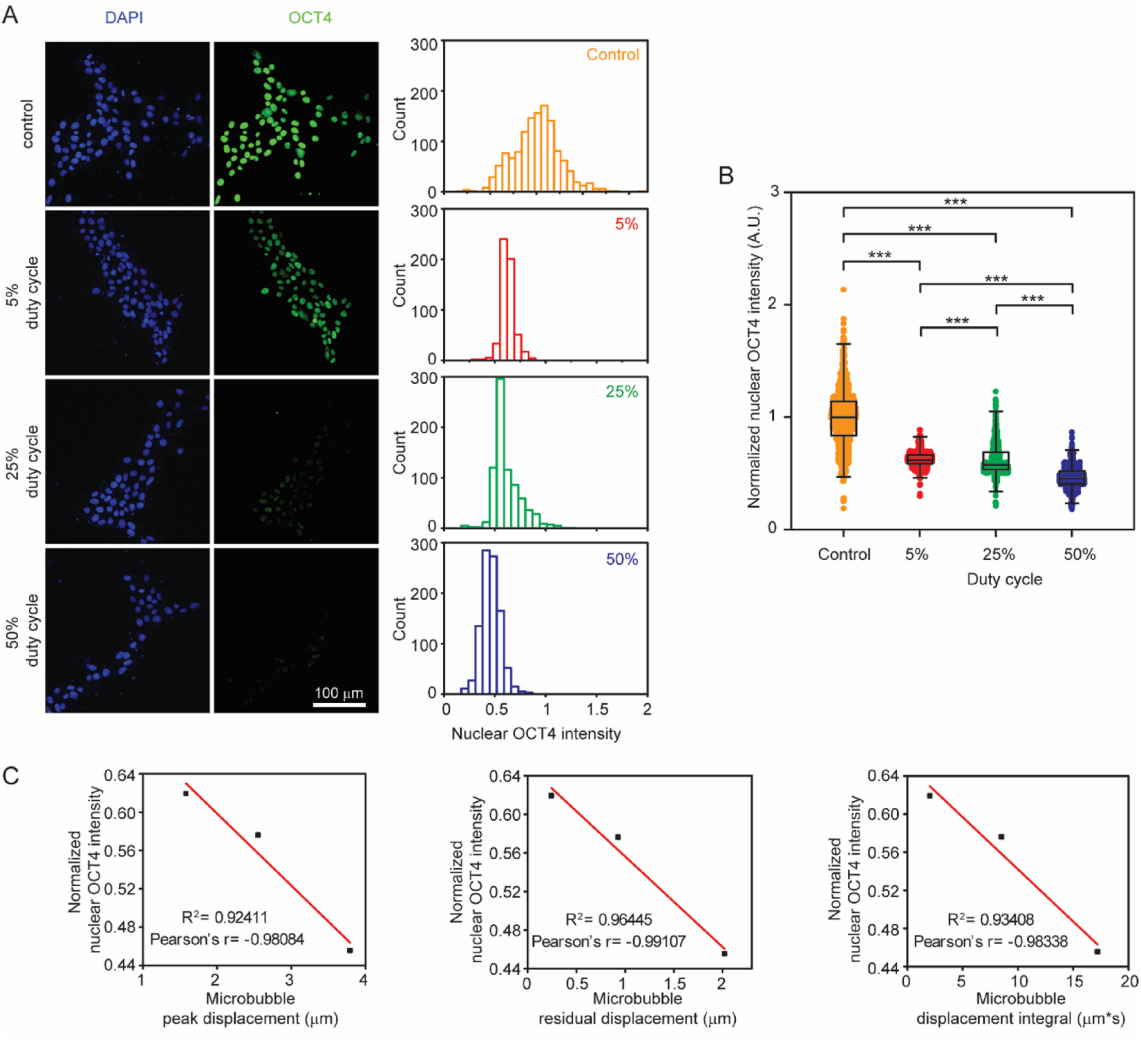
(**A**) Confocal i mages and histograms of immunostaining of OCT4 for cells with and without treatment of ATC with different duty cycles. (**B**) OCT4 fluorescence intensity in cells with and without ATC treatment with different duty cycles. Box: 25–75%, bar-in-box: median, and whiskers: 1% and 99%. *p < 0.05, **p < 0.01, ***p < 0.001. (**C**) Correlations between the median value of peak displacement, residual displacement, and displacement integral with the median value of OCT4 intensity in cells treated by ATC with duty cycle of 5%, 25%, and 50%.

As shown in Fig. 5, applications of ATC significantly downregulated OCT4 and the effect increased with increasing ATC duty cycle (Fig. 5A,B). The OCT4 fluorescent intensity decreased from the control (1.001 ± 0.007) to 0.624 ± 0.003, 0.621 ± 0.005, and 0.462 ± 0.003 for duty cycle of 5%, 25%, and 50% respectively. OCT4 fluorescent intensity was negatively correlated with bubble peak displacement, residual displacement, and displacement integral (Fig. 5C), suggesting that higher strains induced in the microbubble-integrin-CSK linkage might be responsible for pluripotency loss in hPSCs treated by ATC. OCT4 intensity in hESCs treated by ATC at 50% duty cycle, 0.462 ± 0.003, in these experiments was lower than those generated by 30 min ATC treatment without ramping of the acoustic pressure previously (0.756 ± 0.003) (Fig. 2A,B), supporting the effectiveness of ramped acoustic pressure in ATC to compensate bubble size decreases.

## Conclusion

Results in this study demonstrate notable mechanosensitivity of hPSCs to ATC treatments via cyclic deformations of integrin-CSK linkage. Rapid and sustained downregulation of OCT4 and YAP nuclear translocation in hPSCs were detected immediately after ATC stimulations as short as 1 to 5 min. Real time tracking of microbubble displacements during ATC revealed the viscoelastic nature of the cellular integrin-CSK linkage and the role of its deformation in regulating OCT4 and YAP in hPSCs influenced by ultrasound pulse duty cycle or pulse duration. These findings provide new information of the early biophysical effects of ATC on hPSCs and may help the future development of ATC for stem cell differentiation applications.

### Supplementary Information

Supplementary Figures.

## Data Availability

Data included in this manuscript is available and will be shared upon request. Contact Cheri Deng (cxdeng@umich.edu) for data request.
