# Supplementary material for: Rapid responses of human pluripotent stem cells to cyclic mechanical strains applied to integrin by acoustic tweezing cytometry
Source: Sci Rep. 2023 Oct 21;13:18030. doi: 10.1038/s41598-023-45397-5 (PMC10590420; doi:10.1038/s41598-023-45397-5)
Supplement: Supplementary file 1 — Supplementary Figures. [file 41598_2023_45397_MOESM1_ESM.pdf]

**Rapid Responses of Human Pluripotent Stem Cells**

**to Cyclic Mechanical StrainsApplied to Integrin by Acoustic Tweezing Cytometry**

Zhaoyi Xu[a, #], Shiying Liu[b, #], Xufeng Xue[a, #], Weiping Li [b],

Jianping Fu[a,b,c *], and Cheri X. Deng[a,b *]

## Supplementary Figures

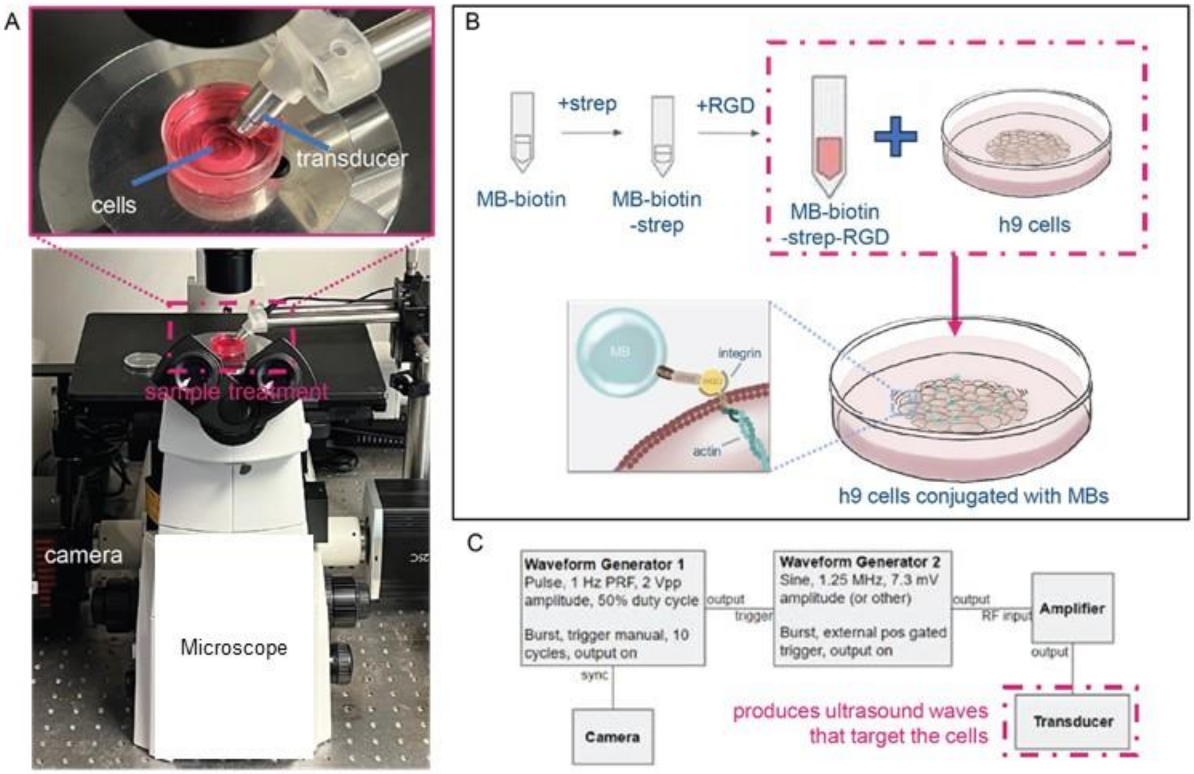


**Figure S1**. (**A**) Representative photos of ATC experimental set-up. (**B**) Microbubble Conjugation Procedures (**C**) Electronic equipment setup for ATC.

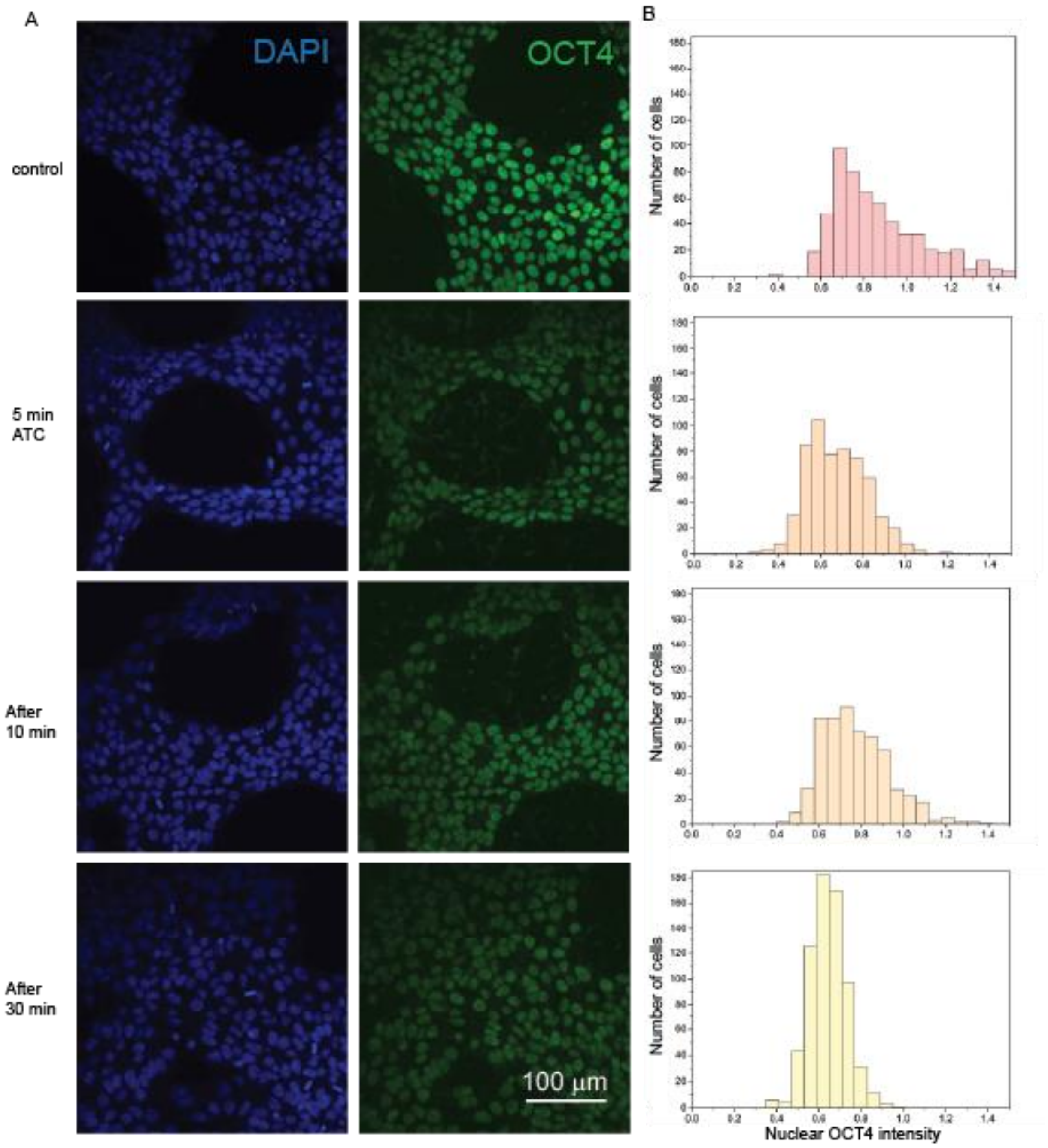


**Figure S2**. (**A**) Representative immunostaining images showing OCT4 expression in hPSCs immediately after ATC treatment, 15.0 min, and 30.0 min post-ATC, respectively. Cells in all groups were treated by ATC for 5.0 min. (**B**) Histogram showing OCT4 expression in hPSCs in each group (control, immediately after ATC treatment, 10 min post-ATC, and 25min post-ATC). Data were collected from 9 samples from three independent experiments for each group.

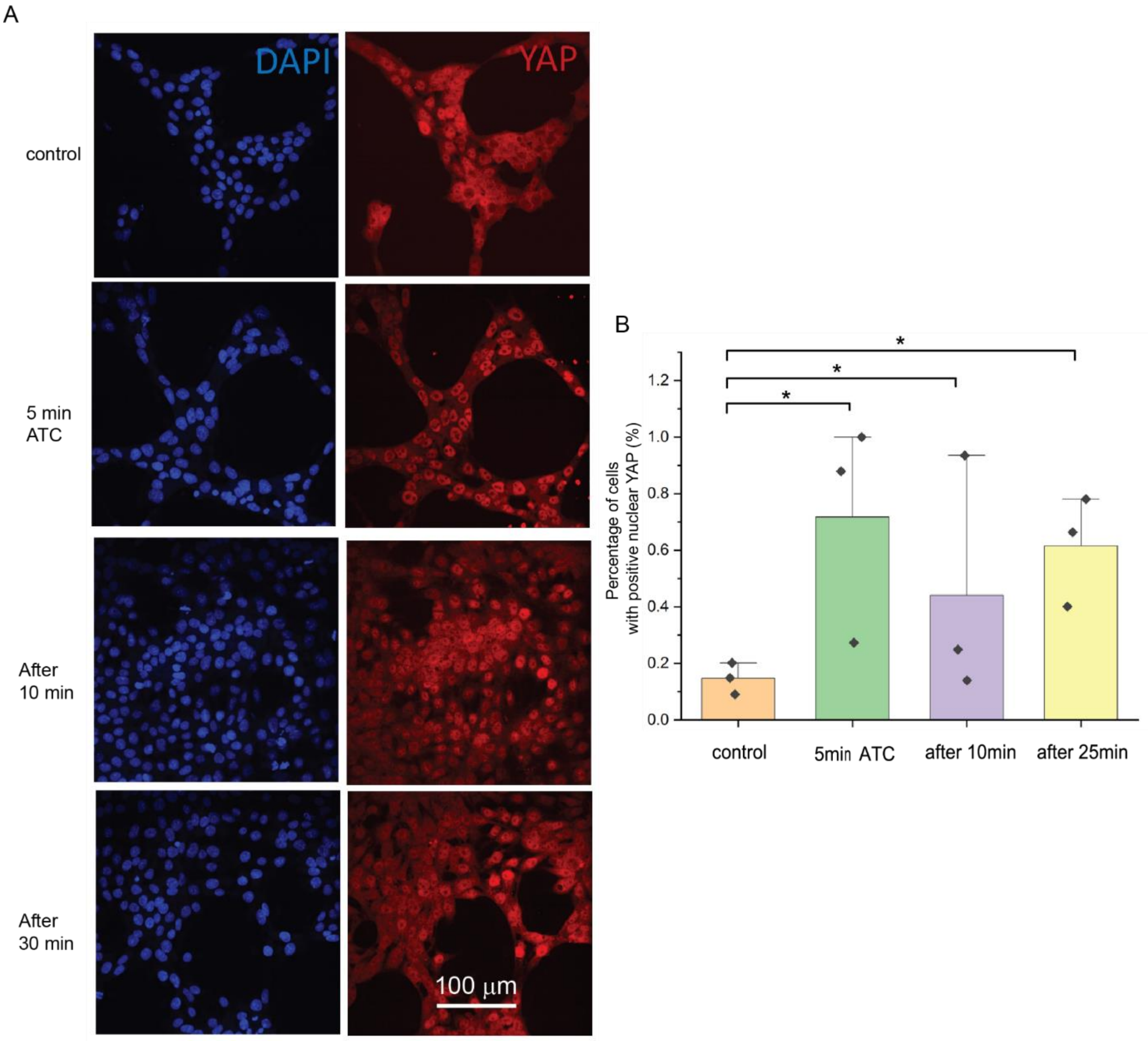


**Figure S3**. (**A**) Representative immunostaining image showing YAP nuclear translocation in hPSCs detected at 5.0 min, 10.0 min, and 25.0 min respectively after 5.0 min ATC treatment. (**B**) Nuclear localization of YAP in hPSCs assayed immediately after 5.0 min ATC treatment, 10 min post-ATC, and 25min post-ATC. Cells in all groups were treated by ATC for 5.0 min.
